# DNA Methylation Dynamics in Blood after Hematopoietic Cell Transplant

**DOI:** 10.1371/journal.pone.0056931

**Published:** 2013-02-22

**Authors:** Ramon M. Rodriguez, Beatriz Suarez-Alvarez, Rubén Salvanés, Manuel Muro, Pablo Martínez-Camblor, Enrique Colado, Miguel Alcoceba Sánchez, Marcos González Díaz, Agustin F. Fernandez, Mario F. Fraga, Carlos Lopez-Larrea

**Affiliations:** 1 Cancer Epigenetics Laboratory, Instituto Universitario de Oncología del Principado de Asturias (IUOPA), HUCA, Universidad de Oviedo, Oviedo, Spain; 2 Department of Immunology, Hospital Universitario Central de Asturias, Oviedo, Spain; 3 Department of Immunology, Hospital Virgen de la Arrixaca, Murcia, Spain; 4 Unidad de Apoyo a la Investigación CAIBER, OIB, Oviedo, Spain; 5 Department of Hematology, Hospital Universitario Central de Asturias, Oviedo, Spain; 6 Servicio de Hematología y Hemoterapia, Hospital Universitario de Salamanca, Salamanca, Spain; 7 Department of Immunology and Oncology, Centro Nacional de Biotecnologıa/CNB-CSIC, Cantoblanco, Madrid, Spain; 8 Fundación Renal “Iñigo Álvarez de Toledo”, Madrid, Spain; Bellvitge Biomedical Research Institute (IDIBELL), Spain

## Abstract

Epigenetic deregulation is considered a common hallmark of cancer. Nevertheless, recent publications have demonstrated its association with a large array of human diseases. Here, we explore the DNA methylation dynamics in blood samples during hematopoietic cell transplant and how they are affected by pathophysiological events during transplant evolution. We analyzed global DNA methylation in a cohort of 47 patients with allogenic transplant up to 12 months post-transplant. Recipients stably maintained the donor’s global methylation levels after transplant. Nonetheless, global methylation is affected by chimerism status. Methylation analysis of promoters revealed that methylation in more than 200 genes is altered 1 month post-transplant when compared with non-pathological methylation levels in the donor. This number decreased by 6 months post-transplant. Finally, we analyzed methylation in IFN-γ, FASL, IL-10, and PRF1 and found association with the severity of the acute graft-versus-host disease. Our results provide strong evidence that methylation changes in blood are linked to underlying physiological events and demonstrate that DNA methylation analysis is a viable strategy for the study of transplantation and for development of biomarkers.

## Introduction

DNA methylation is an epigenetic regulatory mechanism essential for cellular differentiation processes and maintenance of cell type-specific gene expression patterns [1], [2], [3], [4]. Thus, each cell type possesses a stable and characteristic DNA methylation landscape that defines them. Nevertheless, environmental and physiopathological pressures can provoke DNA methylation changes [5], [6], [7]. DNA hypermethylation in the promoter region of tumor suppressor genes is a common hallmark of cancer and its causal relationship with tumor progression has been clearly established [8], [9]. More recently, alterations in DNA methylation have been observed in numerous diseases such as Lupus [10], Alzheimer’s disease [11], diabetes [12], [13] and rheumatoid arthritis [14]. Modifications in DNA methylation also occur in response to environmental factors such as diet [7]. Hence, a large number of publications have revealed the fundamental link between epigenetic deregulation and human disease and consequently, DNA methylation is gaining increasing importance as a source of biomarkers and promising therapeutic targets [15].

Despite the clinical relevance of epigenetic alterations in human disease, the epigenetic approach to the study of solid organ and bone marrow transplantation has been largely overlooked [16]. DNA methylation plays a critical role during hematopoietic differentiation and allows the generation of cell diversity in the immune system [17]. Moreover, it is essential for the establishment of the specific T helper cells subpopulations [18], [19], regulates the expression of cytolityc genes such as perforins and granzymes in NK and activated T cells [20], and contributes to the functionality of memory T cells [21], [22]. Taken as a whole, DNA methylation dynamics in blood are essential for the development of the immune function and this implies that immunological aspects of organ transplantation are partially linked to epigenetic regulation. Obviously, this is especially relevant in the case of bone marrow and hematopoietic cell transplantation since the donor’s immune system is transferred to the recipient and thus, immunotherapy with epigenetic drugs, such as hypomethylating agents, has been recently studied in hematopoietic cell transplant [23], [24].

In this study, we sought to explore how global epigenetic dynamics in blood are affected after transplantation of hematopoietic cells and whether or not DNA methylation patterns change in response to pathophysiological events. For this purpose, we examined global DNA methylation levels and promoter specific methylation in a cohort of 47 patients before and after allogenic hematopoietic cell transplant (HCT). The results indicated that changes in DNA methylation occurred not only during the normal evolution of the transplanted cells, but also in response to pathology. These findings exposed the clinical significance of epigenetic analysis as a diagnostic tool in the field of transplantation.

## Results

### Analysis of Global DNA Methylation Levels Post-HCT

**Figure 1:**
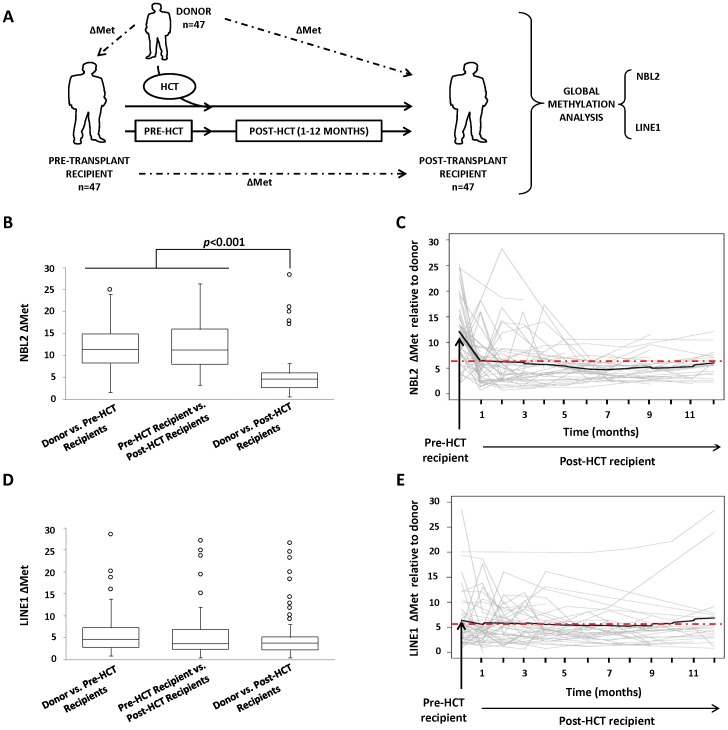
Analysis of global DNA methylation levels post-HCT. (A) Diagram of the experimental design. The differences of the global methylation levels between donors, pre-HCT recipients and post-HCT recipients were assessed by a pyrosequencing based methylation assay of repetitive DNA elements (LINE1 and NBL2) in whole blood. (B) NBL2 ΔMet values between donors, pre-HCT recipients, and 1 month post-HCT recipients. (C) NBL2 ΔMet mean values between donors and recipients up to 12 months post-transplant. The dotted line marks the ΔMet mean value between donors and 1 month post-HCT recipients (ΔMet = 5.8450). During the follow up of the transplant, the mean values barely deviated from the initial post-HCT ΔMet. (D) LINE1 ΔMet values between donors, pre-HCT recipients and 1 month post-HCT recipients. (E) LINE1 ΔMet mean values between donors and recipients up to 12 months post-transplant. The dotted line marked the ΔMet mean value between donors and 1 month post-HCT recipients (ΔMet = 5.383).

In order to study whole genome methylation status, we used pyrosequencing based methylation assay of CpG sites in repetitive DNA elements. Because of the broad distribution of these sequences across the genome, their levels of methylation are considered to be equivalent to the average whole genome methylation. In this study, we analyzed DNA methylation of the LINE1 element which is an interspersed repetitive DNA element that comprises a substantial portion of the genome [25], and the pericentromeric tandem repeat NBL2 [26] in 47 patients after hematopoietic cell transplant by pyrosequencing (Table 1). First, we evaluated the levels of methylation in the donors and in the recipients before and after the transplant and calculated a differential of methylation (ΔMet) that simultaneously compared the methylation values of all CpG sites analyzed in the amplicon between two samples (see details in methods section) (Figure 1A). The ΔMet mean value for the NBL2 between donors and pre-HCT recipients (ΔMet = 12.110) and between pre-HCT recipients and 1 month post-HCT recipients (ΔMet = 12.610) were significantly higher than between donors and 1 month post-HCT recipients (ΔMet = 5.8450) with a *p-value* lower than 0.001 (Figure 1B). These results indicated that the patients retained the NBL2 methylation values found in the donor’s blood at 1 month post-transplant. In addition, long term analysis of NBL2 up to 12 months post-HCT demonstrated that methylation remained consistently similar to the donor (Figure 1C).

**Table 1:** Patient characteristics.

		TOTALN = 47	Non-aGVHD (grade 0)N = 17	aGVHD(grade I)N = 10	aGVHD(grade II–V)N = 20	p-value
**Age Donors (years)**	Median	33	26	35	32	0.966
	Range	(0–63)	(4–58)	(0–52)	(0–62)	
**Age Recipients (years)**	Median	30	30	33	21	0.788
	Range	(1–61)	(1–59)	(7–56)	(6–61)	
**Disease (%)**	Malignant	89% (n = 42)	88% (n = 15)	100% (n = 10)	85% (n = 17)	0.646
	Other	11% (n = 5)	12% (n = 2)	0% (n = 0)	15% (n = 3)	
**Donors type (%)**	Related	66% (n = 31)	76% (n = 13)	80% (n = 8)	50% (n = 10)	0.160
	Unrelated	34% (n = 16)	24% (n = 4)	20% (n = 2)	50% (n = 10)	
**Donor match (%)**	Matched	95% (n = 45)	100% (n = 17)	100% (n = 10)	90% (n = 18)	0.05
	Mismatched	5% (n = 2)	0% (n = 0)	0% (n = 0)	10% (n = 2)	
**Condition regimen (%)**	Moderate intensity	25% (n = 12)	35% (n = 6)	40% (n = 4)	10% (n = 2)	0.170
	High intensity	75% (n = 35)	65% (n = 11)	60% (n = 6)	90% (n = 18)	
**Source (%)**	Peripheral Blood	81% (n = 38)	65% (n = 11)	90% (n = 9)	90% (n = 18)	0.022
	Bone Marrow	15% (n = 7)	35% (n = 6)	0% (n = 0)	5% (n = 1)	
	Umbilical Cord	4% (n = 2)	0% (n = 0)	10% (n = 1)	5% (n = 1)	
**Chimerism**	Complete	79% (n = 37)	53% (n = 9)	100% (n = 10)	90% (n = 18)	0.06
	Mixed	21% (n = 10)	47% (n = 8)	0% (n = 0)	10% (n = 2)	

In case of LINE1, methylation levels were very similar in all samples and no significant differences were observed (Figure 1D). Therefore, LINE1 analysis did not allow a good discrimination of the methylation status between donors and recipients 1 month post-HCT. Nonetheless, LINE1 methylation data from all samples over time (12 months) showed that the average levels of methylation remained stable (Figure 1E) and suggested that there was not a significant epigenetic divergence from the donor and recipient methylation values during the evolution of the transplant in the LINE1 element.

Pyrosequencing analysis of LINE1 and NBL2 only provides limited coverage of the genome so it is possible that other specific genomic regions where these DNA repeats are underrepresented or absent do not follow this trend. To further investigate this issue, we performed a limited methylation study of the subtelomeric repeat D4Z4 in nine patients 1 month after transplant and observed that the ΔMet value between donors and post-HCT recipients (ΔMet = 5.620) was significantly lower than between pre-HCT recipients and post-HCT recipients (ΔMet = 17.930) (Wilcoxon signed-rank test, *p* = 0.003906) (Figure S1). Thus, subtelomeric methylation levels from the donors are dominant over the recipient’s levels when blood samples are analyzed after transplantation, similar to NBL2 methylation analysis.

It is not clear which parameters are affecting NBL2, LINE1 or D4Z4 methylation. It has been observed previously that age, gender and race/ethnicity may affect DNA methylation in repetitive elements [27], [28]. Hence, we performed a limited study with blood samples from a healthy population (n = 90). We could not observe a clear correlation of NBL2 and LINE1 methylation with age (Pearson correlation coefficient, p>0.05) or gender (Wilcoxon, p>0.05) (data not show), although when an arbitrary cutoff point was set at 20 years, older individual showed higher levels of methylation in NBL2 and lower in LINE1 (Table S1). In any case, there was not a linear correlation between DNA methylation in DNA repeats and aging and thus, the moderate effect due to aging does not appear to be sufficient to explain the large inter-individual variability observed in NBL2 methylation.

In summary, these results showed that the recipients stably retained the global methylation status of the donors after transplantation.

### Changes in Global Methylation Levels are Associated to HCT Outcomes

Because donor’s NBL2 methylation levels apparently remained stable in the host after transplantation, we wanted to analyze whether these values were affected by the transplant outcome. To this end, we focused on two clinical parameters, mixed chimerism and acute GVHD, during the first month post-transplant.

**Figure 2:**
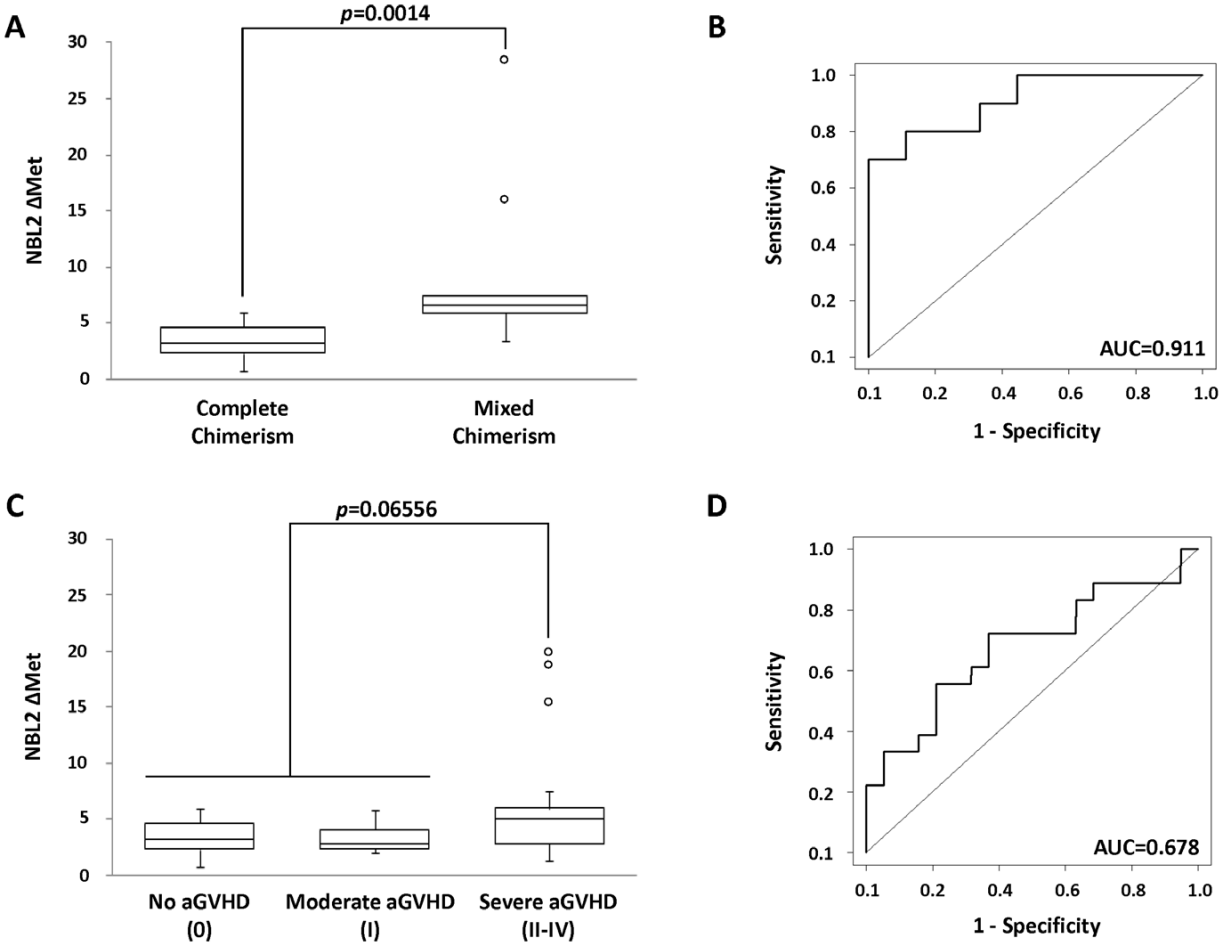
Changes in NBL2 methylation levels are associated to HCT outcomes. (A) NBL2 ΔMet between donors and 1 month post-transplant recipient with complete and mixed chimerism. (B) ROC curve for patients with complete and mixed chimerism (AUC = 0.911). (C) NBL2 ΔMet between donors and 1 month post-transplant recipients according to severity of aGVHD. (D) ROC curve for patients with severe aGVHD versus non-aGVHD and moderate aGVHD (AUC = 0.678).

The control group comprised patients with complete chimerism that did not develop GVHD symptoms during the follow up of the transplant (n = 9). The ΔMet mean value between donors and the recipients 1 month after transplant was higher for patients with mixed chimerism (n = 8, ΔMet = 9.167) than in the control group with complete chimerism (n = 9, ΔMet = 3.400) (Wilcoxon signed-rank test, *p* = 0.0014) (Figure 2A). This result was expected because patients with mixed chimerism had cells from both the donor and the recipient whereas patients with complete chimerism had lost the recipient’s cells. In fact, the inherent sensitivity of NBL2 methylation is notably high, with an area under the ROC (AUC) of 0.911 (Figure 2B). On the other hand, the mean ΔMet value between donors and post-HCT recipients was higher in patients with severe aGVHD (n = 18, ΔMet = 5.870) than in the control group (n = 9, ΔMet = 3.400) or in patients with moderate GVHD symptoms (n = 10, ΔMet = 3.341) (Figure 2C) although it showed a poor discriminative ability (AUC = 0.678) and lacked statistical significance (Wilcoxon signed-rank test, *p* = 0.065) (Fig. 2D).

### Analysis of Promoter Specific DNA Methylation Post-HCT

**Figure 3:**
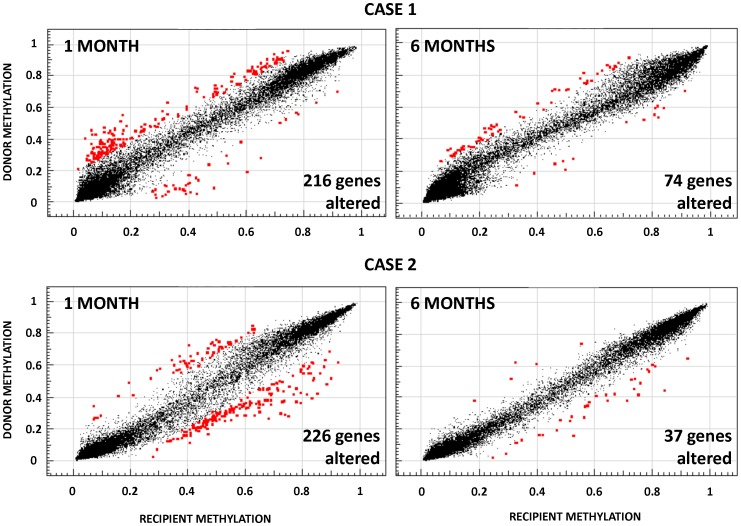
Promoter DNA methylation profiling using bead arrays and differential methylation analysis after HCT. Scatter plots showing DNA methylation in donors versus post-transplant recipients 1 month and 6 months post-HCT. Red dots represent CpG sites with methylation values altered more than 20% relative to donor values.

Since global DNA methylation patterns in post-transplant patients were equivalent to donor values and those values also appeared to change in response to pathophysiological events, we wanted to evaluate DNA methylation in gene promoters. For this purpose, we performed microarray-based DNA methylation profiling in blood samples from a recipient without acute or chronic GVHD (case 1) and from another recipient with grade III aGVHD that evolved into chronic GVHD (case 2). In this analysis, DNA methylation in each probe was considered to be altered when the value differed more than 20% compared to donor. Using this criterion, we found 227 CpG sites (216 genes) with altered methylation values in the case 1 and 238 CpG sites (226 genes) in the case 2 (Figure 3) (Data Set S1), with only 3 genes in common between both samples (DEGS1, TRAF1 and FLJ20273). These differences were also reflected in the gene ontology analysis on the altered genes, showing a differential distribution of biological processes between case 1 and case 2 samples, with a preferential enrichment of GO terms related to immune activation in case 2 (Table S2). On the other hand, 6 months post-HCT, the methylation profile between donor and post-transplant recipient was notably similar, with only 74 altered CpG sites (72 genes) in the case 1 (Figure 3) and 37 (37 genes) in the case 2 (Figure 3) (Data Set S1). These results suggested that methylation profiles in blood samples tend to normalize during the evolution of the transplant.

**Figure 4:**
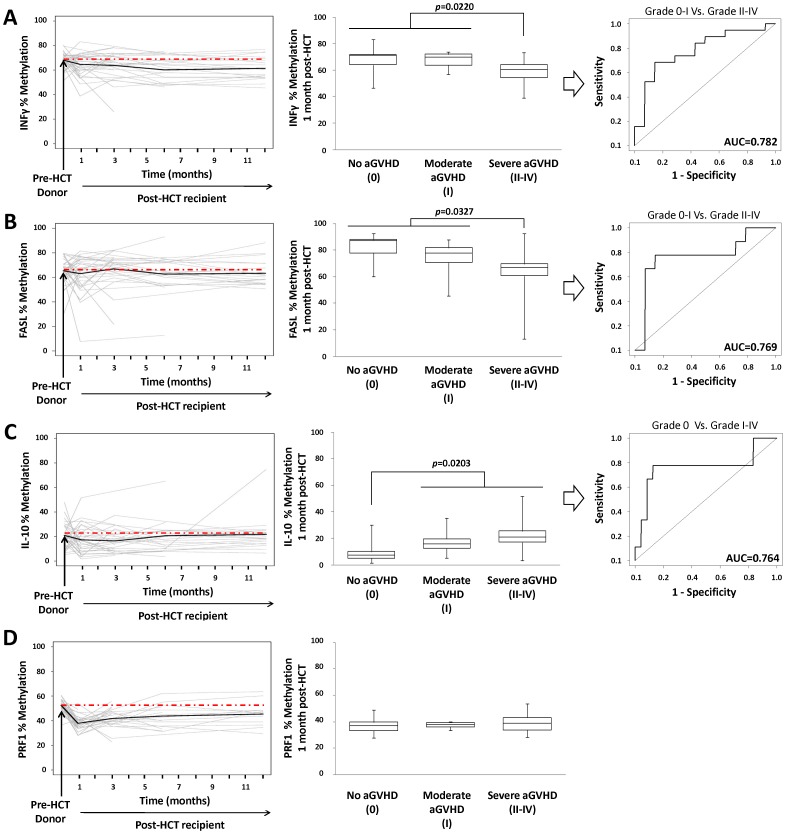
Association of Locus-specific DNA methylation to aGVHD. (A) IFNγ methylation up to 12 months post-transplant is shown in the left panel. The black line marked the mean value in the cohort and the dotted line marked the mean value 1 month post-HCT. In the central panel are represented IFNγ methylation values according to severity of aGVHD 1 month post-HCT. IFNγ ROC curve for patients with severe aGVHD versus non-aGVHD and moderate aGVHD (AUC = 0.782) is shown in the right panel. (B) FASL methylation up to 12 months post-transplant and methylation values according to severity of aGVHD 1 month post-HCT. FASL ROC curve for patients with severe aGVHD versus non-aGVHD and moderate aGVHD (AUC = 0.769) is shown in the right panel. (C) IL-10 methylation up to 12 months post-transplant and methylation values according to severity of aGVHD 1 month post-HCT. IL-10 ROC curve for patient with and without aGVHD (AUC = 0.764) is shown in the right panel. (D) PRF1 methylation up to 12 months post-transplant and methylation values according to severity of aGVHD 1 month post-HCT.

To further explore DNA methylation in gene promoters we selected four genes whose function has been previously associated with the immune response to HCT (IFN-γ, FASL, IL-10, and PRF1) [29], [30], [31] and analyzed their methylation status in a cohort of 47 patients up to 12 months post-HCT. Because DNA methylation in gene promoters is usually associated to gene expression we used in this analysis the real percentage of methylation instead of the ΔMet value. Before transplant, we did not observed differences between donors and recipients in IFN-γ, FASL and IL-10, and only methylation at PRF1 promoter was significantly different (p = 0.0001) (Figure S2). Nonetheless, we observed a large divergence from the donor values starting at 1 month post-HCT and, in case of IL-10 and PRF1, methylation tended to normalize during the evolution of the transplant (Figure 4). Although the average methylation values did not greatly differ overtime, we observed large differences between patients, particularly during the first few months post-HCT, that could be mirroring relevant clinical parameters. We focused in aGVHD 1 month post-HCT because is strongly associated to immune response and typically appears early after transplant. One month post-HCT, methylation levels of IFN-γ and FASL were statistically lower among patients with severe aGVHD (Wilcoxon signed-rank test, *p*<0.05) but were not significant different between the control group and the recipients with moderate aGVHD symptoms (Figure 4A and 4B). The discriminative ability in the case of severe aGVHD is relatively high, with AUC values of 0.782 for IFN-γ and 0.769 for FASL (Figure 4A and 4B). In contrast, IL-10 methylation was higher in patients with aGVHD (Wilcoxon signed-rank test, *p* = 0.0203) with an AUC value of 0.764, but it was not able to discriminate between patients with moderate and severe aGVHD (Wilcoxon signed-rank test, *p* = 0.1541) (Figure 4C). On the other hand, PRF1 methylation values were not significant different between groups 1 month post-HCT (Figure 4D). Finally, the correlation between percentage of methylation and the cellular composition in whole blood was analyzed in a small number of samples for which flow cytometry data was available one month post-HCT (n = 17). Although the sample size was small, we observed statistically significant correlation between the lymphoid fractions and the DNA methylation in some of the analyzed genes (Table S3). These results indicate that DNA methylation is at least partially dependent of the cellular composition in whole blood.

## Discussion

In this study, our aims were to develop a comprehensive epigenetic approach to study hematopoietic cell transplantation. We tackled two parallel parameters: 1) global methylation levels by using pyrosequencing based analysis at repetitive DNA elements of LINE1, NBL2, and D4Z4; and 2) promoter DNA methylation profiling by bead array technology and pyrosequencing at specific gene promoters.

Changes in NBL2, LINE1 and D4Z4 methylation have been reported in cancer [26], [32], [33]. Nonetheless, these global methylation levels can also change in response to environmental factors such as diet, tobacco smoke, or inflammation, and during the natural aging process [7]. Thus, the initial question that we wanted to address is whether or not the interaction between donor cells and the recipient was able to induce global DNA methylation changes in the grafted cells. First, the pericentromeric tandem repeat NBL2 and the subtelomeric element D4Z4 retained the methylation levels of the donor after HCT and, in case of NBL2, analysis up to 12 months post-HCT showed that donor’s global methylation levels remained stable. Therefore, global methylation levels of NBL2 in blood were not significantly affected by the interaction between donor and recipient. LINE1 methylation status was similar between donors and recipients, and importantly, ΔMet values did not change over time. These results further supports the observation that there is not a significant epigenetic drift after transplant in repetitive DNA elements and thus, the epigenetic traits acquired by the donor due to environmental factors or aging are likely to be maintained by the grafted cells in the recipient.

The second question was whether global methylation can change in response to physiological events during HCT. Comparison of NBL2 methylation between patients with complete and mixed chimerism showed that patients with mixed chimerism had a much greater deviation from the donor’s methylation status. Since NBL2 methylation values post-HCT remained stable, this result is easily explained by the mixed contribution of donor and recipient to the blood samples. Global methylation analysis can accurately segregate patients according to chimerism status and therefore transplanted individuals also can be considered chimeric, not only at genetic level, but also at an epigenetic level. Therefore, intrinsic differences found in the NBL2 methylation values between individuals can be used to track down the source of the cells in transplant.

On the other hand, we also observed elevated NBL2 ΔMet values in patients with severe aGVHD which could be due to a real methylation drift as a consequence of the disease. These results suggested that NBL2 methylation could be used as a surrogate marker in HCT. Nonetheless, the data did not reach statistical significance.

In addition to the global DNA methylation analysis during HCT, we wanted to analyze DNA methylation in gene promoters which is often associated with modulation of gene expression. We performed microarray-based DNA methylation profiling in PB samples from two patients with allogenic cell transplant at two different times post-HCT. Methylation profiles post-HCT revealed that the methylation signatures in blood at 1 month post-transplant are markedly altered relative to their donors. Conversely, 6 months post-HCT, the methylation signatures were very similar to the donor suggesting that methylation profiles normalize overtime. Nonetheless, the difference observed between samples at 1 month post-HCT are unlikely to be due to a methylation drift post-HCT since this was not observed at global level by pyrosequencing analysis of repetitive DNA elements. Because we profiled DNA methylation in whole blood, the difference observed at promoter levels probably reflect changes in the cellular composition of the samples and thus normalization of the methylation profile is mirroring the immune reconstitution in the recipient. Each hematopoietic cell type possesses a specific methylation signature [34], [35] and consequently the percent contribution of each cell type to the blood sample results in changes in the methylation profiles. Therefore, methylation analysis could be used to detect alterations of the cellular composition in blood samples if the methylation signature of each cell type was previously defined and consistent in different individuals, although additional studies will be needed to validate this approach.

Additionally, we wanted to explore DNA methylation in gene promoters during transplant evolution in a larger sample set. For this purpose, we examined by pyrosequencing genes typically associated with the immune response to HCT. IFN-γ levels are elevated early during development of GVHD symptoms in both animal models [36] and patients [37], and therefore it constituted a good candidate for validation by pyrosequencing analysis. IFN-γ is an immune activating cytokine whose expression is tightly regulated by DNA methylation. Inverse correlation between DNA methylation and IFN-γ expression has been observed during Th1 differentiation [38] and CD8^+^ lymphocyte activation [39], and it is constitutively hypomethylated in NK cells. [40] Consequently, the hypomethylation at the IFN-γ promoter observed in patients with severe aGVHD suggested enrichment of cytotoxic CD8^+^, NK, and Th1 cells in the blood samples in agreement with an expected expansion of alloreative cells in GVHD. Second, FASL is highly expressed on activated T cells, plays a major role in T-cell mediated cytotoxicity, and thus, it constitutes a key element of the cytolytic response during GVHD in HCT. FASL levels in serum and mRNA levels in CD4^+^ and CD8^+^ T cells are higher among patients with aGVHD. [30], [41] We found that FASL methylation is notably lower in severe aGVHD which is in agreement with the role of FASL in GVHD. Reduced FASL methylation suggests an enrichment of cytolytic cells in the blood samples from these patients, but further studies are necessary since the methylation status of FASL in blood cells has not been clearly established. Thirdly, IL-10 is a cytokine partially regulated by DNA methylation [42], [43] and is mainly expressed by monocytes, Th2 and regulatory T cells which have an inhibitory effect over the expression of Th1 cytokines. [44] Since IL-10 antagonized Th1 response, it was proposed initially that higher levels of IL-10 may be associated with lower risk of GVHD. We found higher methylation levels of IL-10 in blood samples of patients with aGVHD, suggesting that higher methylation levels yield lower levels of IL-10 in aGVHD patients. Nonetheless, IL-10 mRNA in whole blood and protein levels in plasma increased early after HCT in patients with GVHD. [29], [45] This result is only contradictory with our data if we assume that DNA methylation in whole blood is directly related to gene expression. Differential expression profiles in whole blood between two samples could be caused either by changes in the relative cell composition of the sample or by changes of the expression profile of a specific cell type without changes in the relative cellular composition. However, changes of the methylation profiles are more likely due to changes of the cellular composition since DNA methylation patterns are usually stable in terminally differentiated cells. Therefore, it is possible that DNA methylation in whole blood increase because cells with hypermethylation at IL-10 promoter are overrepresented while simultaneously IL-10 levels in plasma increased due to overexpression in response to stimuli in a specific cell type. Taken together, if a particular methylation signature in whole blood is going to be used as a biomarker of disease, it is important to note that DNA methylation levels do not necessarily mirror mRNA levels. Finally, perforin (PRF1) is expressed primarily in NK and CD8^+^ T cells, and is partially regulated by DNA methylation. [20], [31], [46] PRF1 gene is involved in immune mediated cell lysis and mRNA levels are elevated in peripheral blood in patients with aGVHD. [31] We found that PRF1 methylation levels decrease in almost all samples 1 month post-HCT, although we did not observe differences between aGVHD and non-aGVHD patients.

In summary, our results demonstrated that DNA methylation analysis in HCT provides useful information that, with further studies, it may be useful as a diagnostic tool of relevant clinical parameters. The development of robust biomarkers for aGVHD using this strategy will require a much larger discovery set and validation set; nonetheless, our data support the viability of this approach as a source of biomarkers in HCT and paves the way for larger studies. Pyrosequencing-based DNA methylation analysis is relatively inexpensive, is very sensitive, and provides an interesting alternative to protein and mRNA detection in blood samples, allowing the development of novel biomarkers in a large array of human diseases. Further studies will be necessary to fully explore the potential of DNA methylation analysis in the field of hematopoietic cell and solid organ transplantation.

## Methods

### Patients and Samples

DNA samples were obtained from the immunology department of Hospital Virgen de Arraixa in Murcia (Spain) and the Hospital Universitario in Salamanca (Spain) according to approved institutional guidelines. The DNA was obtained from peripheral blood (PB) samples collected monthly from the time of transplant up to 1 year when possible. DNA samples from donors and recipients before transplantation were also obtained. All patients received allogenic hematopoietic cell transplantation and prophylactic pharmacological treatment for graft versus host disease (GVHD). The clinical characteristic of the patients are shown in Table 1. Chimerism was diagnosed by DNA fingerprinting analysis of microsatellites markers (D5S818, Vwa, D13S317, D7S820, D8S1179, D21S11, D3S1358, D18S51, FGA and AMELX/Y) according to institutional guidelines. Diagnosis and grading of acute GVHD (aGVHD) were established as previously described [47]. Additionally, DNA samples from healthy individuals (n = 90) were obtained from the Hospital Universitario Central de Asturias (HUCA). All patients gave their written informed consent in accordance with the declaration of Helsinki and the study obtained the approval by the local ethics committee (Comité Ético de Investigación Clínica Regional Del Principado de Asturias, project number 17/2011).

### Methylation Analysis by Pyrosequencing

Sodium bisulfite modification of 200 ng DNA was carried out with the EZ DNA methylation kit (D5002, Zymo Research, CA, USA) following the manufacturer’s protocol. Pyrosequencing was performed by using the PyroMark kit (Qiagen, Germany). Primers are described in Table S3. The PCR condition was 50 cycles at 95°C for 60 s, 58°C for 30 s, and 72°C for 30 s, followed by 72°C for 5 min. The biotinylated PCR product was purified, made single-stranded, and acted as a template in a pyrosequencing reaction by using the Pyrosequencing Vacuum Prep Tool (Qiagen, Germany). Methylation levels were quantified by using the PyroMark Q24 system (Biotage, Sweden). Six CpG sites were analyzed in NBL2 and D4Z4 sequences and four in LINE1. Primers for IFN-γ, FASL, IL-10 and PRF1 were designed to analyze one CpG site within the promoter region of each gene. Primers are described in Table S4.

### Differential Methylation Analysis by ΔMet Method

The differential of methylation (ΔMet) was developed to allow accurate evaluation of the global methylation drift between two samples by using pyrosequencing based methylation analysis in repetitive DNA elements. The ΔMet was calculated as the Euclidian squared distance in n-space: d(p,q) = ((p_1_ − q_1_)^2^+ (p_2_ − q_2_)^2^+ …+(p_n_ − q_n_)^2^)^0.5^. By this method, each CpG site within the amplicon was treated as a spacial dimension and the methylation values in each CpG site defined a coordinate in an n-space. Therefore, n equaled the number of CpG sites in the amplicon. The distance between the coordinates obtained from two samples was the differential of methylation (ΔMet). This method allowed a very precise assessment of the methylation drift given that it was not affected by non-informative CpG sites or by opposite methylation drift between CpGs within the same amplicon. In addition, the ΔMet value comprised the methylation drift between two samples in a single value, facilitating statistical analysis and data plotting.

### Promoter DNA Methylation Profiling using Bead Arrays and Differential Methylation Analysis

Microarray-based DNA methylation profiling was performed on blood samples from a patient without acute or chronic GVHD and those from a patient with grade III aGVHD that evolved into chronic GVHD. Samples analyzed for each case included donor pre-HCT and recipient samples at 1 month and 6 months post-HCT. Bisulfite conversion of DNA was performed using the EZ DNA Methylation Kit (Zymo Research, CA, USA) according to manufacturer’s procedures. Processed DNA samples were hybridized to the HumanMethylation27 DNA Analysis BeadChip (Illumina, San Diego, CA) and the arrays were scanned on the Illumina iScan system. Raw data were imported and analyzed with the BeadStudio software (version 3.1.3.0 Illumina, Inc) and methylation values were obtained as described [48]. Probes with detection *p*-values of greater than 0.01 were excluded from the analysis. In this study, DNA methylation after HCT in each specific probe was considered to be altered when the value differed more than 20% relative to the donor value. The raw microarray data have been submitted to the NCBI Gene Expression Omnibus (GEO) (http://www.ncbi.nlm.nih.gov/geo/) under de accession number GSE36832. Gene ontology (GO) analysis was performed with the DAVID GO Web-based tool [49], [50]. Redundant GO terms with identical gene hits were excluded in Supporting Information Table 2.

### Statistical Analysis

Analysis was performed with R.2.10 statistical software (www.r-project.org). Due to the asymmetry of the variables, the median was considered the measure of central tendency for statistical calculations. The Wilcoxon test was used to compare the difference between variables. The discriminative ability of the variables was described by receiver operating characteristic (ROC) curves and summarized by the area under the curve (AUC) values. P-values lower than 0.05 were considered significant.

## Supporting Information

Figure S1 Analysis of D4Z4 DNA methylation levels post-HCT. D4Z4 ΔMet values between donors, pre-HCT recipients, and 1 month post-HCT recipients.
(TIF)

Figure S2 Promoter DNA methylation analysis in donors and recipients before HCT. Methylation values were measured by pyrosequencing analysis in pre-HCT samples. Significant differences were assessed by the Wilcoxon signed-rank test.
(TIF)

Table S1 Association between age and global DNA methylation in blood samples.
(DOC)

Table S2 Gene ontology analysis 1 month post-HCT.
(DOC)

Table S3 Correlation between DNA methylation and lymphoid levels measured by flow cytometry in blood samples 1 month post-HCT (n = 17) (*p<0.05, **p<0.01).
(DOC)

Table S4 Primer sets for pyrosequencing and sequences to analyze.
(DOC)

Data Set S1 Promoter DNA methylation profiling using bead arrays and differential methylation analysis after HCT.
(XLS)
