# Correction: DNA Methylation Dynamics in Blood after Hematopoietic Cell Transplant

**DOI:** 10.1371/annotation/2f9880f8-6b33-4b78-9914-d1129a5af8cd

**Published:** 2013-09-20

**Authors:** Ramon M. Rodriguez, Beatriz Suarez-Alvarez, Rubén Salvanés, Manuel Muro, Pablo Martínez-Camblor, Enrique Colado, Miguel Alcoceba Sánchez, Marcos González Díaz, Agustin F. Fernandez, Mario F. Fraga, Carlos Lopez-Larrea

In the Funding Statement, a funding organization was incorrectly listed as providing one of the grants used in the research for the article. In addition, a funding organization and an additional grant were incorrectly omitted from the Funding Statement. The Funding Statement should read: "This work was supported by grants from the Spanish Fondo de Investigaciones Sanitarias-Fondos FEDER European Union (FIS PI08/0566 and FIS P12/02587) and Red de Investigacion Renal (REDinREN). The funders had no role in study design, data collection and analysis, decision to publish, or preparation of the manuscript." 

