# Supplementary figures and images for: DNA Methylation Dynamics in Blood after Hematopoietic Cell Transplant
Source: PLoS One. 2013 Feb 22;8(2):e56931. doi: 10.1371/journal.pone.0056931 (PMC3579934; doi:10.1371/journal.pone.0056931)

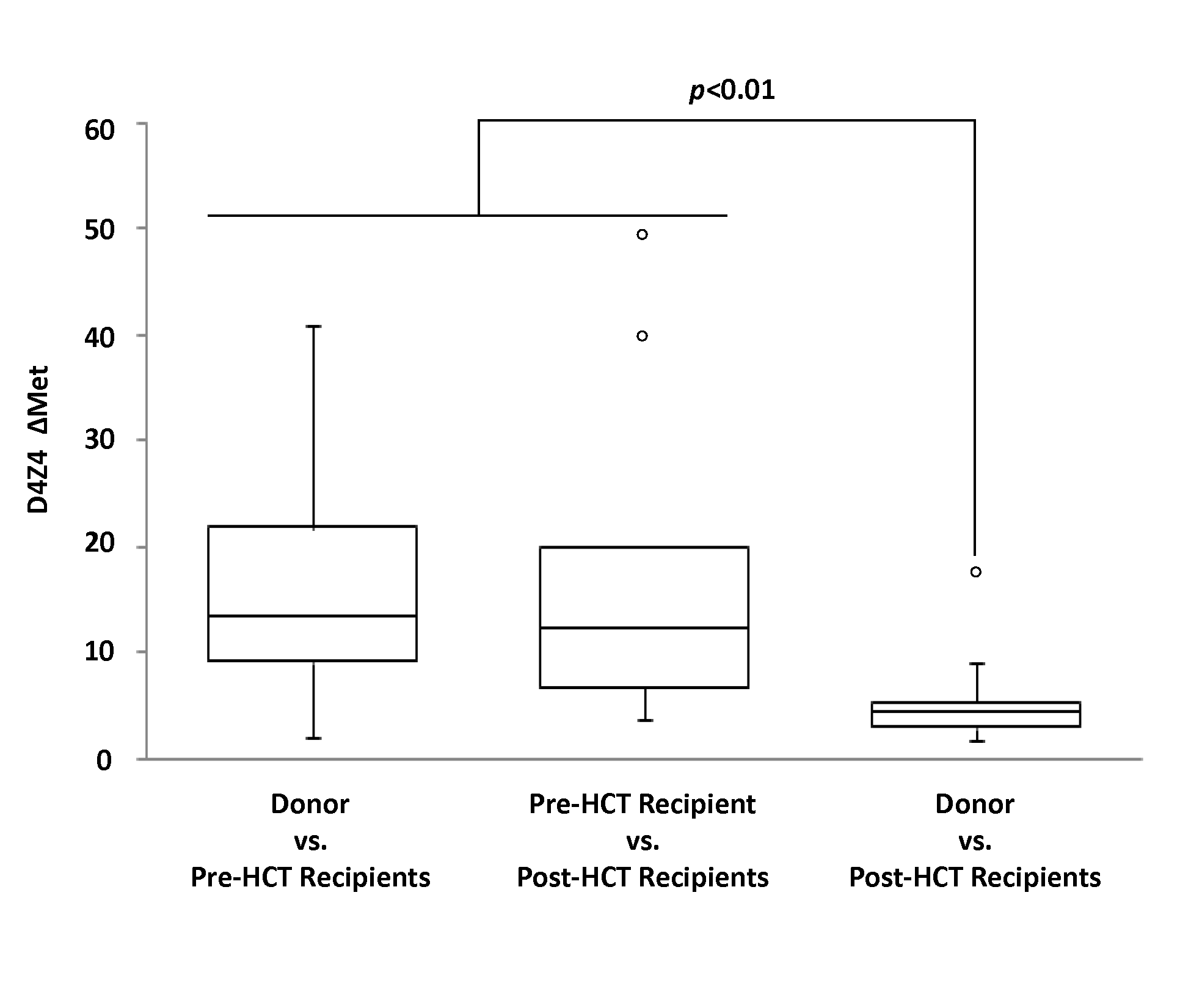

Supplement: Figure S1 — Analysis of D4Z4 DNA methylation levels post-HCT. D4Z4 ΔMet values between donors, pre-HCT recipients, and 1 month post-HCT recipients. (TIF) [file pone.0056931.s001.tif]

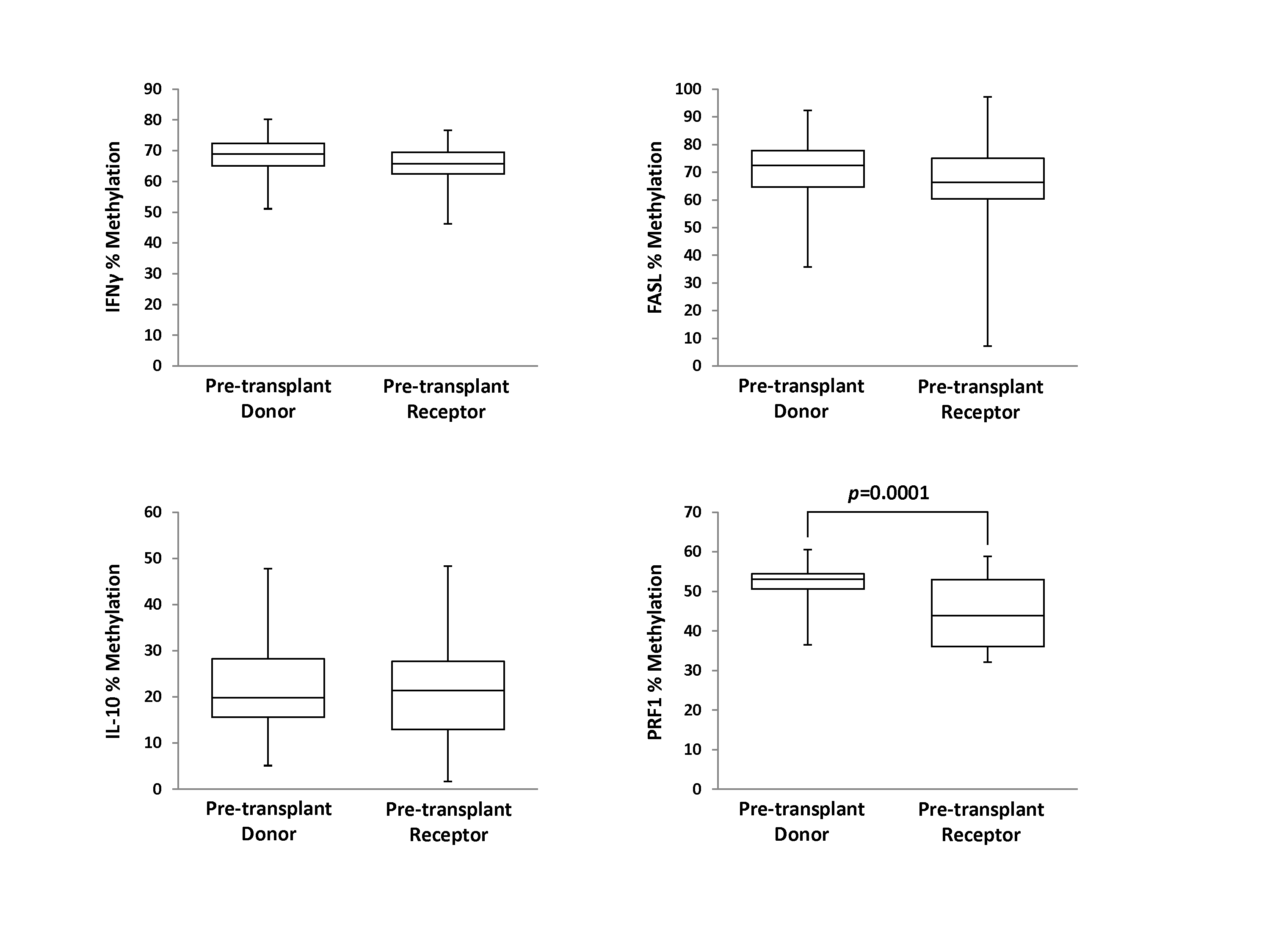

Supplement: Figure S2 — Promoter DNA methylation analysis in donors and recipients before HCT. Methylation values were measured by pyrosequencing analysis in pre-HCT samples. Significant differences were assessed by the Wilcoxon signed-rank test. (TIF) [file pone.0056931.s002.tif]
