# Supplementary material for: DNA Methylation Dynamics in Blood after Hematopoietic Cell Transplant
Source: PLoS One. 2013 Feb 22;8(2):e56931. doi: 10.1371/journal.pone.0056931 (PMC3579934; doi:10.1371/journal.pone.0056931)
Supplement: Table S1 — Association between age and global DNA methylation in blood samples. (DOC) [file pone.0056931.s003.doc]

| **Table S1.** Association between age and global DNA methylation in blood samples. | | | | | | | |
| --- | --- | --- | --- | --- | --- | --- | --- |
| **Amplicon** | **AGE (years)** | **N** | **Mean** | **Median** | **Minimun** | **Maximun** | **P-value (Wilcoxon)** |
| **NBL2** |  |  |  |  |  |  |  |
| CpG 1 | ≤20 | 33 | 82.18 | 82.28 | 74.03 | 90.69 | 0.021 |
|  | 21+ | 57 | 84.36 | 84.63 | 76.26 | 91.57 |  |
|  | Total | 90 | 83.56 | 83.18 | 74.03 | 91.57 |  |
| CpG 2 | ≤20 | 33 | 78.55 | 79.12 | 71.64 | 85.55 | 0.002 |
|  | 21+ | 57 | 80.94 | 81.44 | 73.29 | 86.51 |  |
|  | Total | 90 | 80.06 | 80.51 | 71.64 | 86.51 |  |
| CpG 3 | ≤20 | 33 | 89.51 | 89.36 | 83.65 | 93.34 | 0.005 |
|  | 21+ | 57 | 90.72 | 91.60 | 82.25 | 94.07 |  |
|  | Total | 90 | 90.28 | 90.51 | 82.25 | 94.07 |  |
| CpG 4 | ≤20 | 33 | 89.37 | 89.41 | 81.65 | 100 | 0.017 |
|  | 21+ | 57 | 90.44 | 90.71 | 84.08 | 95.36 |  |
|  | Total | 90 | 90.05 | 90.22 | 81.65 | 100 |  |
| CpG 5 | ≤20 | 33 | 77.01 | 77.95 | 61.61 | 84.29 | 0.105 |
|  | 21+ | 57 | 79.25 | 79.55 | 69.82 | 88.38 |  |
|  | Total | 90 | 78.43 | 79.34 | 61.61 | 88.38 |  |
| CpG 6 | ≤20 | 33 | 67.41 | 68.25 | 51.13 | 80.71 | 0.434 |
|  | 21+ | 57 | 68.53 | 69.48 | 43.12 | 86.93 |  |
|  | Total | 90 | 68.12 | 68.47 | 43.12 | 86.93 |  |
| **LINE1** |  |  |  |  |  |  |  |
| CpG 1 | ≤20 | 33 | 82.19 | 82.09 | 79.16 | 87.29 | 0.021 |
|  | 21+ | 57 | 81.25 | 80.92 | 77.06 | 89.08 |  |
|  | Total | 90 | 81.59 | 81.25 | 77.06 | 89.08 |  |
| CpG 2 | ≤20 | 33 | 52.02 | 54.15 | 40.63 | 57.73 | 0.016 |
|  | 21+ | 57 | 49.92 | 50.97 | 31.91 | 58.54 |  |
|  | Total | 90 | 50.69 | 52.75 | 31.91 | 58.54 |  |
| CpG 3 | ≤20 | 33 | 69.51 | 70.56 | 58 | 79.65 | 0.006 |
|  | 21+ | 57 | 66.84 | 68.68 | 57.59 | 74.24 |  |
|  | Total | 90 | 67.82 | 69.48 | 57.59 | 79.65 |  |
| CpG 4 | ≤20 | 33 | 54.42 | 55.85 | 46 | 62.71 | 0.049 |
|  | 21+ | 57 | 52.87 | 52.12 | 42.85 | 67.72 |  |
|  | Total | 90 | 53.44 | 54.98 | 42.85 | 67.72 |  |
