# Supplementary material for: DNA Methylation Dynamics in Blood after Hematopoietic Cell Transplant
Source: PLoS One. 2013 Feb 22;8(2):e56931. doi: 10.1371/journal.pone.0056931 (PMC3579934; doi:10.1371/journal.pone.0056931)
Supplement: Table S2 — Gene ontology analysis 1 month post-HCT. (DOC) [file pone.0056931.s004.doc]

| **Table S2**. Gene ontology analysis 1 month post-HCT. | | | |
| --- | --- | --- | --- |
| **Sample** | **GO Terms with the highest statistical significance** | **Gene Count** | **P-value** |
|  |  |  |  |
| **Case 1** | Circulatory system process | 10 | 3.35E-04 |
|  | Hemoglobin metabolic process | 3 | 0.005736 |
|  | Erythrocyte development | 3 | 0.008285 |
|  | Transmembrane receptor protein tyrosine kinase signaling pathway | 8 | 0.01616 |
|  | Tube morphogenesis | 6 | 0.016601 |
|  | Enzyme linked receptor protein signaling pathway | 10 | 0.019042 |
|  | Regulation of body fluid levels | 6 | 0.024828 |
|  | Response to hormone stimulus | 10 | 0.028389 |
|  | Morphogenesis of an epithelium | 5 | 0.030565 |
|  | Response to peptide hormone stimulus | 6 | 0.034091 |
|  |  |  |  |
| **Case 2** | Defense response | 31 | 1.70E-11 |
|  | Immune response | 29 | 5.69E-09 |
|  | Chemotaxis | 11 | 1.71E-05 |
|  | Defense response to bacterium | 9 | 4.72E-05 |
|  | Response to bacterium | 11 | 8.45E-05 |
|  | Response to wounding | 18 | 1.42E-04 |
|  | Inflammatory response | 13 | 4.09E-04 |
|  | Leukocyte chemotaxis | 5 | 8.56E-04 |
|  | Locomotory behavior | 11 | 0.001355 |
|  | Positive regulation of cytokine secretion | 4 | 0.00229 |
|  |  |  |  |
