# Supplementary material for: DNA Methylation Dynamics in Blood after Hematopoietic Cell Transplant
Source: PLoS One. 2013 Feb 22;8(2):e56931. doi: 10.1371/journal.pone.0056931 (PMC3579934; doi:10.1371/journal.pone.0056931)
Supplement: Table S3 — Correlation between DNA methylation and lymphoid levels measured by flow cytometry in blood samples 1 month post-HCT (n = 17) (*p<0.05, **p<0.01). (DOC) [file pone.0056931.s005.doc]

| **Table S3**. Correlation between DNA methylation and lymphoid levels measured by flow cytometry in blood samples 1 month post-HCT (n=17) (* p<0.05, ** p<0.01). | | | | | |
| --- | --- | --- | --- | --- | --- |
|  | | **FASL** | **IFN-γ** | **IL-10** | **PRF1** |
| **LT CD4^+^** | Pearson Correlation  P value | 0.455  0.066 | 0.484  0.049 (*) | -0.307  0.230 | 0.148  0.663 |
| **LT CD8^+^** | Pearson Correlation  P value | -0.473  0.055 | -0.543  0.024 (*) | 0.276  0.284 | -0.781  0.005 (**) |
| **NKs** | Pearson Correlation  P value | 0.168  0.520 | 0.187  0.472 | -0.240  0.354 | 0.371  0.261 |
| **LB** | Pearson Correlation  P value | 0.062  0.813 | 0.193  0.458 | 0.360  0.156 | 0.286  0.377 |
