# Supplementary material for: DNA Methylation Dynamics in Blood after Hematopoietic Cell Transplant
Source: PLoS One. 2013 Feb 22;8(2):e56931. doi: 10.1371/journal.pone.0056931 (PMC3579934; doi:10.1371/journal.pone.0056931)
Supplement: Table S4 — Primer sets for pyrosequencing and sequences to analyze. (DOC) [file pone.0056931.s006.doc]

| **Table S4**.Primer sets for pyrosequencing and sequences to analyze. | | |
| --- | --- | --- |
| **Amplicon** | **PCR primers (5'-3')** | **Sequencing primers (5'-3') and analyzed sequences** |
| **NBL2** | Fw: AGTAGTTGGTGTTAATGTGTGTT | Sq: TGGTGTTAATGTGTGTTAT |
| (214 bp) | Rv: Biotin-AAACCTCTTTACTCCTCTAATAAAC | *YGGAGGTATAYGGGGYGAYGGYGAAAYGAAGGGTGGGGTTTAGGTATATGTYGGT* |
| **LINE1** | Fw: TTTTTTGAGTTAGGTGTGGG | Sq: GGGTGGGAGTGAT |
| (248 bp) | Rv: Biotin-TCTCACTAAAAAATACCAAACAA | *TYGATTTTTTAGGTGYGTTYGTTATTTTTTTTTTTGATTYGGAAAGGGAA* |
| **D4Z4** | Fw: GGTGGTTYGGGGTAGGG | Sq: GGGAATATTTGGTTGGTTA |
| (175 bp) | Rv: Biotin-CCCAAAAAAAAATAACAATTCTC | *YGGAGGGGYGTGTTTTYGTTTYGTTTTTTTTATYGGGTTGATYGGTTTGGGAT* |
| **INFγ** | Fw: GGATTTAAGGAGTTTAAAGGAAATT | Sq: ATTATTTTATTTTAAAAAATTTGTG |
| (180 bp) | Rv: Biotin- ACACCTCCTCTAACTACTAATATTTATAC | *AAAATAYGTAATTTTTAGGAGATTTTAATTAGGTATAAATATTAGTAGTTAGAGGAGGTGT* |
| **FASL** | Fw: TGGGTGTTTTTTTGAGAAGTAGA | Sq: GAGTAGTTAGTAATAGGGT |
| (208 bp) | Rv: Biotin-TAATAAATCAAACCAACCCCAACA | *TTYGTTTTTGATATTTTAGTTTTTATAGGATTGAGAAGAAGTAAAATYGTTTG* |
| **IL-10** | Fw: Biotin-ATTTTATTGTATTTTGGAATGGGTAATTTG | Sq: CCTCTCTAATAAACTTAATTTTCAA |
| (272 bp) | Rv: TCCTCCTTCTCTAACCTCTCTAATA | *TTTTTACATCRTAAACAAAAATAATTAATTAAACATAAACTTCTACATTACAACTATTTTT* |
| **PRF1** | Fw: GAGGTTTTTATGGGTGGAGTGAT | Sq: TTGGGGGGTAAAATT |
| (91 bp) | Rv: Biotin-CACCTCCTCCCTTACCCAACTA | *ATAYGGTTTTT* |
